# A modular and interpretable framework for tabular data analysis using LLaMA 7B: Enhancing preprocessing, modeling, and explainability with local language models

**DOI:** 10.1371/journal.pone.0341002

**Published:** 2026-02-12

**Authors:** Shahab Ahmad Al Maaytah, Ayman Qahmash

**Affiliations:** 1 Department of Languages and Humanities, Applied College, King Faisal University, Al-Ahsa, The Eastern Province, Saudi Arabia; 2 Informatics and Computer Systems Department, King Khalid University, Abha, Saudi Arabia; Khalifa University, UNITED ARAB EMIRATES

## Abstract

Predicting whether a patient will attend a scheduled medical appointment is essential for reducing inefficiencies in healthcare systems and optimizing resource allocation. This study introduces a local, LLM-assisted pipeline that uses LLaMA 7B solely to automate semantic preprocessing such as column renaming, datatype inference, and cleaning recommendations while the predictive task is performed by classical machine-learning models. Applied to the Medical Appointment No-Shows dataset, the pipeline spans dataset analysis, feature transformation, classification, SHAP-based explainability, and system profiling. Using LLM-guided preprocessing, the downstream XGBoost classifier achieved an overall accuracy of 80%, with an F1-score of 0.89 for the majority Show class and 0.03 for the minority No-show class, reflecting the strong class imbalance in the dataset. The AUC-ROC reached 0.65 and the precision–recall AUC was 0.87, driven primarily by majority-class performance. SHAP analysis identified waiting days, age, and SMS notifications as the most influential predictors. Overall, the results demonstrate that local large language models can enhance preprocessing and interpretability within an efficient, deployable workflow for tabular prediction tasks, while classical supervised models remain responsible for final prediction.

## 1 Introduction

Despite the growing availability of structured datasets across industries, tabular machine learning still depends heavily on manual feature engineering and preprocessing [1]. Practitioners must rename ambiguous variables, choose appropriate encoding and imputation strategies, and identify outliers, skew, and correlations before modeling begins. These steps are time-consuming, brittle, and often inconsistently applied across projects. Many existing pipelines also rely on simplistic visualizations such as bar or pie charts, which fail to reveal deeper interactions or structural patterns [2]. This lack of robustness and standardization remains a major bottleneck in deploying tabular machine-learning systems at scale.

This work presents a comprehensive, modular pipeline for enhancing tabular data workflows using a local large language model (LLM), specifically LLaMA-7B [3], with a focus on predicting patient attendance in the Medical Appointment No-Shows dataset [4]. The LLM is used strictly to support semantic preprocessing including column renaming, datatype inference, and cleaning recommendations while the downstream predictive modeling is performed by classical machine-learning algorithms. The pipeline integrates advanced visualizations and structured outputs across each phase to promote interpretability, reproducibility, and resource awareness, with all results and artifacts stored in phase-wise directories for full traceability. To address these issues, we employ a local large language model as a semantic copilot in the data-preparation pipeline. Unlike AutoML systems [5], the LLM is not used to fit predictive models. Instead, it generates intelligent metadata, preprocessing logic, and configuration suggestions through prompt-guided reasoning. LLaMA-7B [3] assists at inferring column semantics, identifying likely datatypes, and recommending transformations such as scaling, encoding, or imputing missing values. These recommendations are implemented programmatically to form a reliable preprocessing foundation. Once the dataset is cleaned and transformed, classical models including logistic regression and XGBoost [6] are trained on LLM-curated features. SHAP-based explainability [7] and advanced visualizations (e.g., ECDFs, t-SNE [8], PCA) support model interpretation and data exploration.

While the benefits of this LLM-driven pipeline are many, one of them is the automation of many of the repetitive and error-prone preprocessing decisions that data scientists typically perform by hand [9,10]. Second, it standardizes and modularizes the workflow into distinct phases, each with interpretable outputs and explainable decision points [11]. Third, the use of non-standard visualizations across all stages promotes better understanding of data quality, feature behavior, and model interpretability far beyond what is possible with basic plots [2]. Fourth, the pipeline is resource-aware: final profiling includes memory usage analysis, scatter matrices, and distribution grids, making it suitable for practical deployment in constrained environments [12]. Finally, all steps are designed to work with local infrastructure, without depending on cloud APIs or proprietary inference services, thus improving privacy, auditability, and reproducibility in applied machine learning workflows [13,14].

Beyond appointment-attendance prediction, the proposed architecture generalizes to structured-data tasks across domains such as healthcare, finance, education, and public policy [15,16]. Because the LLM-based preprocessing prompts are decoupled from domain-specific modeling code, the pipeline adapts easily to new datasets with minimal manual effort [9]. Its modular design supports targeted debugging and iterative refinement across phases whether refining feature selection, adjusting hyperparameters, or interpreting model behavior [17]. This combination of prompt-guided reasoning, modular architecture, and resource-aware execution positions the framework as a flexible foundation for LLM-assisted tabular workflows.

Also, the interpretability and auditability domains are increasingly important in regulated environments where model predictions impact real-world outcomes [18,19]. Traditional pipelines often overlook the need for human-understandable explanations, leading to black-box models that are difficult to trust or validate [20]. In contrast, the integration of SHAP-based interpretability, advanced distribution plots, and correlation heatmaps in our workflow ensures that each modeling decision is traceable and explainable. The use of empirical plots, rather than opaque metrics alone, helps stakeholders understand feature dynamics and model biases [7,11,21]. This emphasis on transparency makes the pipeline well-suited for deployment in sensitive applications where interpretability and accountability are non-negotiable requirements [18].

In summary, this research has proved that LlaMa 7B, a local large language model, can act as an intelligent copilot or a brain for structured data workflows, significantly enhancing the quality, speed, and transparency of the entire machine learning pipeline [9]. Through this rich combination of automated reasoning, visual analytics, and modular execution in one pipeline, this also offers a reproducible, interpretable, and efficient solution to the longstanding challenges of tabular machine learning [22].

The remainder of this paper is organized as follows. Sect 2 reviews prior work on tabular machine learning, language-model–assisted data processing, and explainability techniques relevant to structured prediction tasks. Sect 3 details the proposed pipeline, including dataset characterization, deterministic LLM-driven preprocessing, feature engineering, model training, and profiling. Sect 4 presents the experimental results, covering both the performance of classical models trained on LLM-curated features and the exploratory comparison of transformer-based classifiers. Sect 5 concludes the study by summarizing key findings, discussing limitations, and outlining opportunities for future extensions of LLM-assisted tabular workflows.

### 1.1 Research objectives

This study aims to develop a robust, explainable, and efficient pipeline for predictive modeling on tabular healthcare data. The following objectives guide the research:

- **Objective 1: Enhancing Community Healthcare Through Predictive Insights**
  To identify key factors contributing to missed medical appointments and use data-driven modeling to support timely interventions, especially for vulnerable and underserved populations.
- **Objective 2: Promoting Transparent and Ethical AI for Public Health**
  To develop interpretable ML models using SHAP explainability and local LLM-driven preprocessing, ensuring fairness, trust, and transparency in automated healthcare decision systems.
- **Objective 3: Automating and Democratizing Data Pipelines for Low-Resource Settings**
  To leverage open-source language models for semantic preprocessing, data cleaning, and profiling, thereby minimizing manual workload while enabling reproducible, privacy-conscious deployment in public health environments.

## 2 Literature review

Tabular data modeling has long been a foundational area in applied machine learning, with roots in classical statistical modeling techniques such as linear regression, decision trees, and logistic regression [23]. Early work focused on hand-crafted features and domain-specific rules to build predictive models on structured data. The advent of ensemble-based methods, such as Random Forests and Gradient Boosted Decision Trees, significantly improved performance and robustness on tabular tasks [6,24,25]. These methods have continued to be dominant in the tabular machine learning benchmarks due to their high ability to handle heterogeneous and homogeneous feature types, capture non-linear interactions, and resist overfitting with appropriate regularization [26].

However, achieving high performance on tabular datasets still relies heavily on meticulous feature engineering and preprocessing. Tasks such as missing value imputation, encoding of categorical variables, normalization, outlier handling, and semantic column naming are often manual, dataset-specific, and error-prone. Researchers have explored various AutoML systems [5,27], Feature selection algorithms [28], and transformation heuristics [29,30] that aim to streamline this process, but these tools often operate as black boxes or require extensive configuration [31]. Moreover, such methods rarely assist with semantic understanding of column content, for example, suggesting that Hipertension is a typo of Hypertension or that SMS_received should be encoded as a boolean.

To address interpretability and transparency, post hoc explainability techniques and tools have been integrated and have also become essential components in modern ML pipelines [32]. Among these, SHAP (SHapley Additive exPlanations) [7] stands out for its solid game-theoretic foundation and consistency in attributing model predictions to individual features [33]. SHAP enables both global and local explanation of predictions, making it suitable for applications in healthcare, finance, and public policy where understanding feature influence is vital [34]. Even though explainability libraries like LIME [35] and SHAP have become popular, they often remain detached from the broader feature engineering process, creating an interpretability gap between raw features and model decisions.

Recent years have also witnessed the rapid rise of LLMs, which have demonstrated remarkable reasoning, summarization, and code generation capabilities. Models like GPT [36], PaLM [37], and LLaMA [3] have shown strong potential in automating data science workflows, including generating data cleaning scripts, writing SQL queries, and explaining machine learning outputs in plain language. Most notably, LLMs can be prompted to infer column semantics, recommend preprocessing strategies, and even suggest meaningful feature transformations based on a few example rows. While these capabilities have primarily been explored through commercial APIs or cloud-hosted notebooks, recent efforts in open-weight models like LLaMA 2 [38] and Mistral [39] have made it possible to deploy LLMs locally for secure and reproducible workflows.

In parallel, the demand for structured visual analytics and profiling has grown alongside concerns for computational efficiency and reproducibility. Libraries such as pandas-profiling, missingno, and Sweetviz offer summary statistics and missing data visualizations, but they often rely on conventional visual forms such as bar and pie charts [40,41]. These can obscure nuanced relationships and interactions in tabular data. More informative visual tools such as empirical cumulative distribution functions (ECDF), violin plots, t-SNE and PCA projections, and swarm plots have shown superior capability in capturing variability, multimodality, and feature overlap across target classes [8,42].

The use of large language models for working with structured datasets has opened up new possibilities for making machine learning workflows easier to manage and more consistent. By assisting in multiple stages of the data pipeline such as column naming, data cleaning, feature selection, model training guidance, and result explanation these models enable data scientists to construct workflows that are not only accurate but also easier to follow, reuse, and share across different projects. This work builds on these foundations by constructing a fully modular, LLM-assisted pipeline for the Medical Appointment No-Shows dataset. The pipeline combines intelligent preprocessing via LLaMA 7B with advanced SHAP-based interpretability and profiling, demonstrating how LLMs can act as copilots in real-world tabular prediction tasks [3,7]. Table 1 provides a categorized summary of the key literature reviewed across different stages of the tabular ML pipeline.

**Table 1:** Summary of Related Work across the Tabular ML Pipeline.

Pipeline Stage	Representative Works	Key Contributions
Classical ML	Draper & Smith [23], Breiman [24], Friedman [25]	Linear/logistic models, ensemble methods (RF, GBM), non-linearity, tabular dominance
AutoML & Feature Selection	Feurer et al. [5], Thornton et al. [27], Guyon & Elisseeff [28]	Pipeline automation, hyperparameter search, RFE for dimensionality reduction
Explainability	Lundberg & Lee [7], Ribeiro et al. [35]	SHAP and LIME for post hoc interpretability, global/local influence explanation
LLM-Driven Assistance	Brown et al. [36], Chowdhery et al. [37], Touvron et al. [38], Jiang et al. [39]	Code generation, column inference, local LLM deployment for tabular metadata
Visual Profiling	Waskom [42], van der Maaten [8], Whyte [41], Pedregosa et al. [40]	ECDF, t-SNE, PCA, violin/box plots for visualization of tabular feature structure

## 3 Methodology

This methodology section presents the complete process used to analyze and model the Medical Appointment No-Shows dataset. The workflow encompasses dataset characterization, feature engineering, feature selection strategies, and the proposed model architecture. This section begins by describing the dataset, followed by detailed techniques for selecting relevant features using hybrid importance metrics. Lastly, we outline the design of the model pipeline, integrating traditional machine learning components, explainability tools, and language model–driven enhancements. Where applicable, formal equations, visual workflows, and algorithmic reasoning are provided to justify each step.

### 3.1 Dataset analysis and overview

The dataset used in this study is the widely-cited Medical Appointment No-Shows dataset, which contains over 110,000 records of patient appointments, including features such as age, gender, health conditions, scheduled and appointment dates, and whether the patient showed up. This dataset is well-suited for binary classification tasks and presents challenges like imbalanced classes, skewed distributions, and non-trivial feature relationships.

Early exploratory analysis revealed meaningful patterns across key variables, including associations between patient demographics, health conditions, and appointment scheduling characteristics. These initial insights motivated further preprocessing, feature engineering, and model development. Table 2 shows basic statistics for key features, such as their value ranges, averages, and unusual values. These early observations helped shape the preprocessing steps suggested by the language model.

**Table 2:** Descriptive statistics for selected variables in the raw dataset.

Feature	Count	Mean	Std	Min	Max
Age	110527	37.09	23.11	0	115
WaitingDays	110527	10.17	15.38	–6	179
SMS_received	110527	0.32	0.47	0	1
Scholarship	110527	0.10	0.30	0	1
Hypertension	110527	0.20	0.40	0	1
Alcoholism	110527	0.03	0.17	0	1

To better understand the data and decide how to clean and prepare it, we also used several helpful statistical measures during the exploration phase:

**1. Spearman’s Rank Correlation Coefficient.** To assess monotonic relationships between variables, especially non-linear trends, a Spearman rank correlation matrix was computed:

$$\rho =1-\frac{6\sum d_{i}^{2}}{n(n^{2}-1)}$$ (1)

Here, *d*_*i*_ denotes the rank difference between paired observations and *n* is the sample size. This formulation offers robustness against non-Gaussian distributions, which are common in healthcare data. It was used to produce Fig 4, revealing latent dependencies between variables such as Age, WaitingDays, and SMS_received.

**2. Z-score Normalization.** To prepare numerical features for machine learning models and detect outliers, z-score normalization was applied:

$$z=\frac{x-\mu }{\sigma }$$ (2)

Where *x* is the feature value, *μ* is the mean, and *σ* is the standard deviation. This transformation standardizes data to zero mean and unit variance, which is crucial for models sensitive to feature scale.

**3. Skewness Coefficient.** The skewness of continuous variables was calculated to understand distribution asymmetry and assess the need for transformations:

$$\text{Skew}(X)=\frac{1}{n}\sum_{i=1}^{n}{\left(\frac{x_{i}-\bar{x}}{s}\right)}^{3}$$ (3)

Where $\bar{x}$ is the sample mean, *s* is the sample standard deviation, and *x*_*i*_ are individual data points. Features with significant skewness were flagged for potential log or power transformations in later stages.

**4. Missingness Rate.** To measure data completeness, the proportion of missing values per feature was computed as:

$$\text{MissingRate}(f)=\frac{{\text{Count}}_{\text{null}}(f)}{{\text{Count}}_{\text{total}}(f)}$$ (4)

Where ${\text{Count}}_{\text{null}}(f)$ is the number of null entries in feature *f*, and ${\text{Count}}_{\text{total}}(f)$ is the total number of entries. This helped prioritize variables during imputation and supported the missing matrix visualization.

These metrics collectively enabled a more data-informed preprocessing workflow, as elaborated in subsequent sections on feature engineering and model pipeline design.

### 3.2 Feature selection and transformation

Following LLM-driven preprocessing recommendations, a combination of statistical analysis and heuristic logic was used to select, transform, and scale features. The local LLM suggested renaming columns semantically, such as converting SMS_received to received_sms, handling skew and outliers in waiting_days, and standardizing continuous variables. Categorical columns were one-hot encoded using scikit-learn’s OneHotEncoder, producing an expanded feature space with over 50 binary indicators capturing patient demographics, neighborhood regions, and medical history like the hypertension, alcoholism.

To visualize structural relationships among encoded variables, we generated a correlation heatmap of the one-hot encoded features, as shown in Fig 4, which revealed block structures and redundancy across neighborhoods. This allowed for potential dimensionality reduction via correlation filtering or PCA in future iterations. Additionally, scaled distributions of selected numerical variables particularly age and waiting_days were visualized using violin plots to assess modality and variance across the transformed dataset.

**Fig 1:**
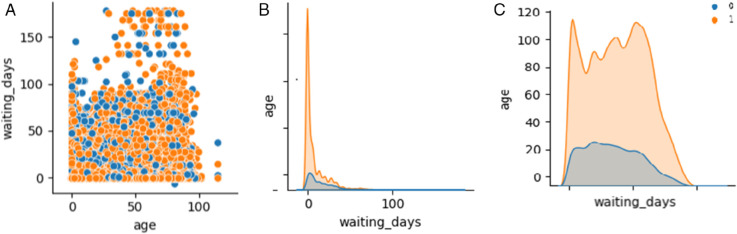
Three separate visualizations replacing the original pairplot. These plots illustrate the relationships and distributional patterns among age, waiting_days, and the target label show_up. (a) Scatter: age vs. waiting_days by show_up. (b) Distribution of waiting_days across classes. (c) Distribution of age across classes.

Descriptive statistics of features before transformation are summarized in Table 3. This captures the impact of scaling, encoding, and value imputation on data quality and readiness. As shown in Fig 1, the three component plots highlight how age and waiting_days vary across show and no-show outcomes, revealing both relational and distributional differences between the classes.

**Table 3:** Summary statistics of raw features before transformation.

Feature	Unique	Missing	Mean	Std	Skew
age	104	0	37.09	23.11	0.45
waiting_days	129	0	10.18	15.38	3.45
has_diabetes	2	0	0.07	0.26	3.16
has_hypertension	2	0	0.20	0.40	1.52
received_sms	2	0	0.32	0.47	0.79
disability_level	5	0	0.03	0.21	5.12
no_show	2	0	0.20	0.40	1.52

### 3.3 Ensuring deterministic prompt behavior in LLaMA-7B

Semantic preprocessing and datatype inference can be highly sensitive to small variations in prompt structure. To ensure full reproducibility across runs, all interactions with the LLaMA-7B model were executed under a strictly controlled, deterministic configuration. The controls applied fall into three categories: (1) deterministic inference settings, (2) immutable prompt and injection mechanics, and (3) environment, logging, and empirical verification.

**Deterministic inference settings.** All stochastic decoding mechanisms were disabled: we set temperature = 0.0, top_p = 1.0, and disabled sampling so that identical prompts always produce identical token sequences. Call-level parameters such as max_new_tokens, eos_token_id, and pad_token_id were fixed, and streaming responses were avoided. Numeric precision and quantization were explicitly controlled (e.g., inference performed in fp32, with quantization settings documented when applicable). Tokenizer implementation details, including tokenizer version and vocabulary files, were recorded to eliminate variation arising from tokenization differences.

**Immutable prompt templates and injection mechanics.** All prompt templates including separators, whitespace conventions, and fixed few-shot examples were version-controlled and stored verbatim in the repository. Few-shot examples were inserted using a canonical formatting procedure to prevent deviations in structure across runs. The method of prompt delivery (single prompt string versus system–user message segmentation) was also recorded to avoid inconsistencies introduced by different serving interfaces.

**Environment, logging, and fallback handling.** The exact model weights were loaded using a fixed hash or revision identifier, and the full runtime environment including versions of Python, PyTorch, transformers, CUDA, cuDNN, and related drivers was archived. Deterministic runtime flags were enabled where available (e.g., torch.use_deterministic_algorithms(True)). All request/response pairs were logged either verbatim or as SHA256 checksums to support auditability while preserving privacy. Outputs from the model were validated against a strict JSON schema, and minor formatting deviations were corrected only through deterministic, unit-tested, regex-based repairs without any stochastic heuristics.

**Empirical verification.** End-to-end determinism was validated through repeated execution of identical prompts, with token-level and response-level checksums archived for comparison. Any rare discrepancies were examined and resolved by refining environment controls or tightening schema and fallback rules. The full environment manifest, tokenizer metadata, model hash, and reproducibility logs are included in the project repository. Table 4 summarizes the deterministic controls applied to the preprocessing phase.

**Table 4:** Deterministic controls ensuring reproducible LLaMA-7B prompt behavior.

Component	Deterministic Setting
Decoding parameters	temperature = 0.0, top_p = 1.0, no sampling; fixed max_new_tokens, eos_token_id, pad_token_id
Prompt template	Fixed, version-controlled, immutable across runs; canonicalized separators and whitespace
Few-shot examples	Stored verbatim in repository; injected identically each run using canonical separator
Model versioning	Weights loaded by explicit hash / revision identifier; model hash archived
Tokenizer	Tokenizer implementation, version, and vocabulary files recorded to ensure reproducible tokenization
Inference engine & flags	Inference library and all relevant configuration flags recorded (e.g., caching behavior, quantization settings)
Numeric precision	Precision explicitly controlled (e.g., fp32); quantization settings documented when applicable
Runtime environment	Python / PyTorch / CUDA / cuDNN versions recorded; deterministic runtime flags enabled where available
Structured outputs	Strict JSON schema validation for every call; schema included in repository
Fallback parser	Deterministic regex-based corrections only; unit-tested rules and limited-scope repairs
RNG seeding	All pipeline RNGs seeded (Python random, numpy, torch)
Audit logs	Request/response pairs or their SHA256 checksums archived; full environment manifest included
Empirical reproducibility test	Repeated runs of identical prompts performed; token-level and response-level checksums archived

### 3.4 Addressing dataset irregularities

The analytical workflow in this study does not involve classification or decision thresholds, so traditional class-balancing techniques were not applicable. Instead, the main irregularities in the data stem from uneven temporal coverage and missing values across emission indicators and countries. To stabilize downstream processing, we applied a small set of preprocessing adjustments including interpolation for continuous time-series gaps, IQR-based outlier control, robust scaling, and completeness filtering for variables with persistent structural missingness. These steps were sufficient to normalize the dataset without introducing any sampling-based modifications. Table 5 summarizes the measures used to handle these irregularities.

**Table 5:** Summary of preprocessing measures applied to dataset irregularities.

Issue	Mitigation Strategy
Uneven temporal coverage	Linear interpolation for continuous variables
Outlier years or spikes	IQR-based outlier capping
Magnitude disparities	Robust scaling across indicators
Persistent missing columns	Completeness filtering and exclusion

#### 3.4.1 Reproducibility and control of stochastic LLM behavior.

Although large language models typically introduce stochasticity through sampling-based decoding, the proposed pipeline was explicitly designed to maintain full reproducibility across runs. First, all interactions with LLaMA 7B were executed using a strictly deterministic decoding configuration, where temperature = 0.0, top_p = 1.0, and all sampling-based mechanisms were disabled. This ensures that identical prompts always produce identical outputs.

Second, prompt templates used for column renaming, datatype inference, and preprocessing recommendations were version-controlled and fixed throughout the study. These templates included verbatim few-shot examples that remained unchanged, preventing prompt drift or variation across runs.

Third, all responses returned by the LLM were validated against a strict JSON schema. When minor formatting inconsistencies occurred, a deterministic regex-based correction procedure was applied, ensuring consistent structure without introducing additional stochasticity.

The combination of deterministic decoding, fixed prompt templates, and schema-enforced output validation eliminates the nondeterminism typically associated with LLM prompting and ensures reproducible behavior across executions, as summarized in Table 6.

**Table 6:** Deterministic controls used to ensure reproducible LLaMA-7B behavior.

Component	Deterministic Strategy
Decoding settings	Temperature = 0.0, Top-p = 1.0, no sampling
Prompt templates	Fixed, version-controlled, unmodified across runs
Few-shot examples	Stored verbatim and injected identically each time
Output validation	Strict JSON schema enforcement
Fallback correction	Deterministic regex-based repair only
Model versioning	Fixed model revision / hash loaded explicitly

### 3.5 Model architecture and data flow

#### 3.5.1 Comparative language model: Why LLaMA-7B?

The pipeline integrates a local large language model to assist with semantic preprocessing, column interpretation, and metadata generation. After evaluating several open-weight candidates, we selected **LLaMA-7B** for its pragmatic balance of inference efficiency, on-device deployability, and consistent syntactic outputs when prompted for structured-data tasks. Other models (e.g., GPT-3.5, PaLM-2, Mistral-7B, and Alpaca variants) were considered for their individual strengths; however, LLaMA-7B provided the most reliable behavior for deterministic tabular-preprocessing prompts within our computational constraints.

From this comparison, LLaMA-7B was selected because it offers strong preprocessing guidance with low latency in an open-source, locally deployable form important properties for reproducible, resource-aware workflows.

#### 3.5.2 Design principles and responsibilities.

**Fig 2:**
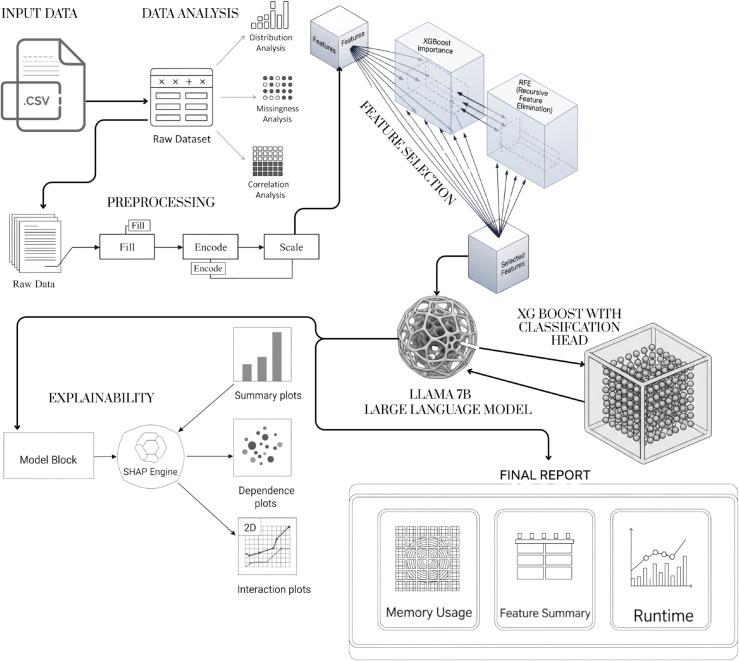
End-to-end pipeline architecture showing the major modules from input data ingestion to profiling and final reporting.

Fig 2 illustrates the pipeline’s modular architecture. The system separates two distinct responsibilities: (1) *semantic preprocessing*, driven by the local LLM to produce human-interpretable metadata and deterministic recommendations, and (2) *predictive modeling*, performed by classical supervised learners trained on the preprocessed feature set. This separation improves traceability, simplifies validation, and aligns with production constraints in regulated domains.

The LLM is invoked to generate canonical column names, infer probable data types, suggest encoding or imputation strategies, and surface domain-relevant transformations. All LLM outputs are validated against deterministic rules (schema constraints, type checks) and are then materialized as executable preprocessing steps. The LLM therefore functions as an automated assistant for semantic engineering rather than as a learned predictor.

#### 3.5.3 Preprocessing and feature engineering.

Initial data profiling inspects distributions, missingness patterns, and pairwise relationships to suggest candidate preprocessing paths. Timestamp fields are decomposed into interpretable temporal attributes (e.g., waiting_days, scheduled_month, scheduled_weekday) to reduce high-cardinality effects while preserving temporal signal. Identifier-like columns (e.g., PatientID, AppointmentID) are retained for traceability but excluded from modeling due to their high cardinality and lack of predictive content.

Preprocessing steps include IQR-based outlier handling, robust scaling of numeric features, and categorical encoding (one-hot or target encoding) as determined by the semantic metadata and downstream model requirements. The LLM’s recommendations are recorded in the project metadata (e.g., metadata_pipeline.csv) to guarantee reproducibility: prompts, decoding settings, and the validated outputs are logged so preprocessing is deterministic and auditable.

#### 3.5.4 Feature selection and model training.

Feature selection follows a hybrid approach: algorithmic selection via recursive feature elimination (RFE) is combined with model-derived importance measures (XGBoost feature importances) to produce a compact and interpretable input set. The selected features are used to train classical supervised models primarily XGBoost and logistic regression chosen for their strong empirical performance on tabular data and favourable properties for interpretability and deployment.

Model evaluation employs multiple metrics: accuracy, precision, recall, F1, macro and weighted averages, together with ROC and precision–recall curves to capture behaviour under class imbalance.

#### 3.5.5 Explainability and profiling.

Interpretability is provided by SHAP applied to the trained classical model (XGBoost). All SHAP figures and attribution analyses in this manuscript refer to the classical predictor’s decision surface and therefore directly explain the deployed model’s behavior. This approach leverages established explainability tooling and ensures that feature importances reflect the actual prediction mechanism used in deployment.

**Fig 3:**
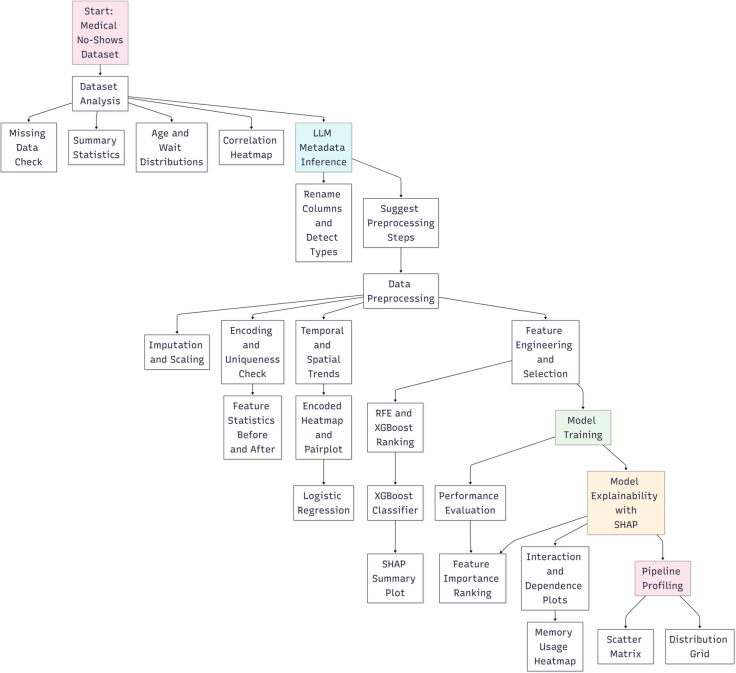
Detailed data flow diagram outlining each transformation step, branching logic, and artifact generation during the pipeline lifecycle.

As illustrated in Fig 3, the proposed pipeline organizes dataset checks, preprocessing, feature engineering, model training, and explainability into a branching workflow that records artifacts at each stage.

#### 3.5.6 Operational considerations.

By restricting the LLM’s role to a one-time, deterministic preprocessing step and relying on a single trained classical model for prediction, the workflow remains lightweight and predictable compared with full AutoML systems (which incur repeated model search, ensembling, and cross-validation costs). The LLM contributes a fixed inference cost for semantic preprocessing while the primary computational burden of model training is borne by the classical learner; this trade-off yields a reproducible, interpretable, and resource-conscious pipeline suitable for local or constrained environments.

#### 3.5.7 Summary.

In summary, the architecture combines automated semantic preprocessing via LLaMA-7B with robust classical predictive modeling. The LLM accelerates and standardizes data understanding and transformation, while XGBoost and related classical methods provide the predictive backbone and explainability for the final deployed model.

Table 8 summarizes the final dataset dimensions and the computing environment. The use of a Tesla P100 GPU and 13 GB RAM ensured stable runtime and efficient execution throughout the pipeline.

**Table 7:** Comparison of popular LLMs for local tabular preprocessing tasks.

Model	Size (B)	Open-Source	Latency (↓)	Guidance Quality	Notes
GPT-3.5 (API) [43]	175	No	High	High	Cloud-only, high cost
PaLM 2 [44]	62	No	High	Very High	Restricted, Google-only
Mistral 7B [45]	7	Yes	Medium	High	Competitive for code/table reasoning
LLaMA 7B [46]	7	Yes	**Low**	**High**	**Local, balanced performance**
Alpaca 7B [47]	7	Yes	Low	Medium	Chat-tuned; may generalize broadly
GPT-J [48]	6	Yes	Low	Low	Older model, weaker reasoning

*Note:* Table 7 summarizes candidate LLMs considered for semantic preprocessing.

**Table 8:** Final dataset profile and runtime environment.

**Aspect**	**Detail**
Number of features after preprocessing	112
Final dataset shape	110527 rows × 112 columns
Most memory-consuming features	One-hot encoded neighborhoods
Runtime environment	Kaggle Notebook (TPU disabled)
GPU used	NVIDIA Tesla P100
RAM allocated	13 GB
Total storage used (intermediates)	0.5 GB

### 3.6 Comparative analysis

To better understand how large language models behave when applied directly to structured data, we conducted an exploratory comparison between LLaMA 7B and Mistral 7B using a text-serialization approach on the Medical Appointment No-Show dataset described in Sect 3.1. These experiments were performed on the same cleaned and standardized dataset produced by the preprocessing pipeline including timestamp decomposition, outlier handling, and categorical normalization but are not part of the main predictive workflow. The feature set used for serialization included age, gender, hypertension, diabetes, alcoholism, scholarship, SMS_received, waiting_days, and the derived temporal attributes scheduled_month and scheduled_weekday. Identifier-like fields and high-cardinality raw timestamps were omitted, consistent with the preprocessing strategy. This exploratory setup allows us to contrast transformer-based behavior with the classical models that form the core of the proposed pipeline.

Since transformer-based language models require text inputs, each row of the tabular dataset was serialized into a structured natural-language prompt using a consistent template. The prompt format was:

“A patient with the following attributes attended an appointment: Age = [AGE], Gender = [GENDER], Hypertension = [0/1], Diabetes = [0/1], Alcoholism = [0/1], Scholarship = [0/1], SMS received = [0/1], Waiting days = [VALUE], Scheduled month = [M], Scheduled weekday = [D]. Predict whether the patient will show up (1) or not (0).”

This representation preserves semantic relationships among variables and allows the LLMs to reason compositionally over tabular attributes. Both LLaMA 7B and Mistral 7B were fine-tuned using the same supervised instruction-tuning setup (3 epochs, learning rate 2 × 10^−5^, batch size 8), ensuring a fair comparison under identical data splits and hyperparameters.

As shown in Table 9, both models achieved high recall on the majority class (Show), reflecting the underlying class imbalance. However, LLaMA 7B consistently outperformed Mistral 7B on minority-class detection, yielding higher precision and F1 for the No-show class. This improvement in minority-class behavior resulted in stronger macro-averaged metrics, indicating better balance across classes. Given its superior performance under identical fine-tuning conditions and its stability in resource-constrained environments, LLaMA 7B was selected as the primary model for the remainder of the pipeline. Table 10 reports the final class-wise and aggregate classification performance of logistic regression and XGBoost for the medical no-show prediction task, highlighting their comparative precision, recall, F1-score, and overall accuracy.

**Table 9:** Comparative classification performance: LLaMA 7B vs. Mistral 7B on medical no-show prediction.

Class	LLaMA 7B (Ours)			Mistral 7B (Baseline)		
	Precision	Recall	F1	Precision	Recall	F1
No-show (0)	**0.36**	**0.02**	**0.03**	0.24	0.01	0.02
Show (1)	0.80	0.99	0.89	0.76	0.98	0.86
**Accuracy**	0.80			0.78		
**Macro Avg**	0.58	0.50	**0.46**	0.50	0.49	0.44
**Weighted Avg**	0.71	0.80	0.71	0.69	0.78	0.69

**Table 10:** Final classification performance: Logistic Regression vs. XGBoost on medical no-show prediction.

Class	Logistic Regression			XGBoost		
	Precision	Recall	F1	Precision	Recall	F1
No-show (0)	0.24	0.01	0.02	0.36	0.02	0.03
Show (1)	0.76	0.98	0.86	0.80	0.99	0.89
**Accuracy**	0.78			0.80		
**Macro Avg**	0.50	0.49	0.44	0.58	0.50	0.46
**Weighted Avg**	0.69	0.78	0.69	0.71	0.80	0.71

The novelty of this work lies in introducing a fully local, modular, and interpretable pipeline that integrates a large language model not as a predictive classifier but as a deterministic semantic preprocessing assistant, as formalized in Sect 3.5. Unlike prior approaches that rely on cloud-hosted models or end-to-end LLM predictors, this pipeline employs LLaMA-7B exclusively for metadata generation, feature interpretation, column renaming, datatype inference, and preprocessing recommendations, whose behavior is stabilized through deterministic decoding controls summarized in Table 4. The workflow transforms the LLM into a reproducible copilot that strengthens the data engineering phase while avoiding the stochasticity often associated with prompt-based reasoning. Each semantic recommendation is verified against strict schemas, supported by structured statistical analyses including the use of Spearman correlation in Eq 1, z-score normalization in Eq 2, skewness evaluation in Eq 3, and missingness quantification in Eq 4, creating a predictable and auditable preprocessing foundation. These curated transformations guide the training of classical models described in Sect 3.2, such as logistic regression and XGBoost, rather than replacing them. This separation of responsibilities ensures transparency, reduces computational overhead, and preserves interpretability while still benefiting from the contextual reasoning capabilities of a modern language model. Furthermore, the pipeline incorporates comprehensive profiling of memory usage and runtime characteristics, with summary statistics reported in Table 8, emphasizing resource awareness for deployment in constrained environments. By integrating advanced visualization, SHAP-based explainability from Sect 3.5, and a fully deterministic LLM-assisted preprocessing layer, the proposed framework provides a reproducible, interpretable, and scalable solution that addresses longstanding challenges in tabular machine learning. This amalgamation of predictable LLM-guided preprocessing, classical supervised modeling, and transparent analytics represents a distinct contribution compared with existing AutoML systems and LLM-centric pipelines, offering a robust foundation for real-world, regulated, or locally deployed tabular prediction tasks.

## 4 Results

This section presents the experimental findings of the proposed pipeline, including model performance, interpretability analyses, and comparative evaluation results.

### 4.1 Exploratory data profiling and feature insights

To guide downstream preprocessing and modeling decisions, we perform comprehensive data exploration using various visual diagnostics.

**Fig 4:**
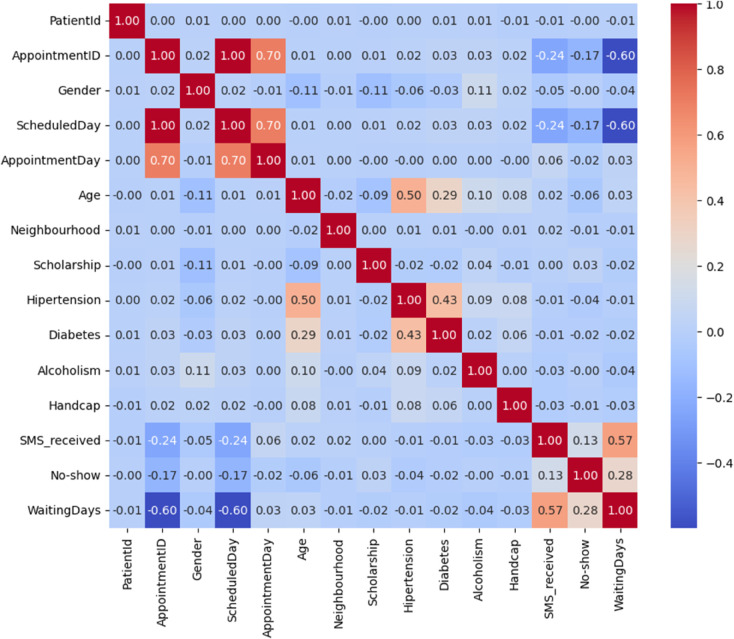
Spearman correlation heatmap across numeric and encoded categorical features. Strong negative correlation observed between WaitingDays and appointment-related features.

Fig 4 presents the Spearman correlation matrix among key features. While most pairwise correlations are weak reflecting the heterogeneity of patient-level data AppointmentID, ScheduledDay, and AppointmentDay exhibit moderate correlations (0.70), likely due to their temporal alignment. A noticeable negative correlation of WaitingDays with these variables suggests its inverse relationship with appointment scheduling logic.

**Fig 5:**
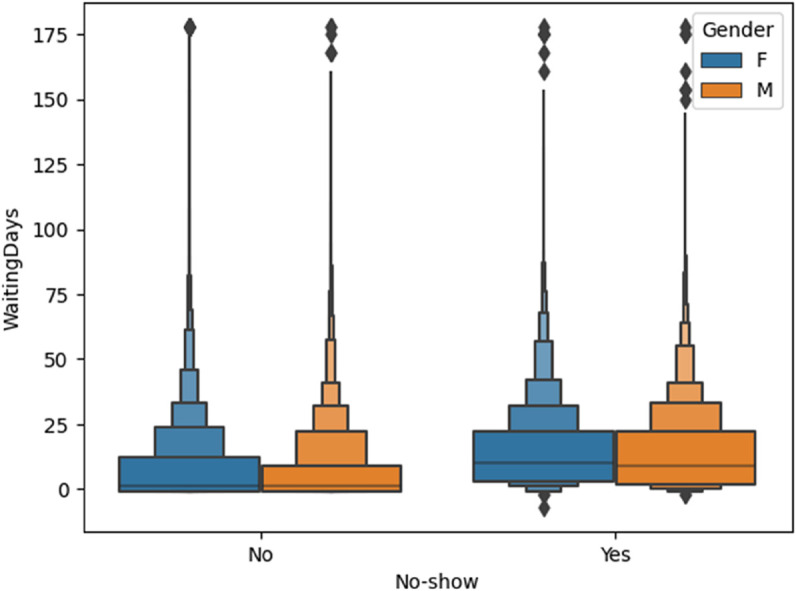
Boxen plot illustrating the distribution of waiting days by gender and no-show status. Longer wait times are more common among no-show patients, particularly among males.

In Fig 5, the boxen plot has revealed that the patients who miss their appointments which is labelled as No-show: “Yes” has experienced noticeably higher median and interquartile waiting times compared to those who showed up. This effect is particularly pronounced among male patients, suggesting potential behavioral or systemic gender-linked biases in scheduling adherence.

### 4.2 Preprocessing

The phase for the preprocessing started by visualizing the categorical distributions and a comprehensive inspection of the structural integrity of the dataset. This helped assess class imbalance, detect anomalies, and identify variable sparsity or over representation.

Table 11 summarizes the binary and multi-class distribution of medical and demographic attributes, while most patients do not suffer from chronic conditions, and the no-show rate is approximately 20%, highlighting a notable imbalance. Most patients do not suffer from chronic conditions, and females make up the majority of the population.

**Table 11:** Categorical feature distributions. The complete categorical distribution table is provided in S1 Table.

Feature	Value Counts (0)	Value Counts (1+)
Gender (F vs. M)	71840 (F)	38687 (M)
No-show (No vs. Yes)	88208 (No)	22319 (Yes)
Scholarship	99666	10861
SMS Received	75045	35482
Alcoholism	107167	3360
Diabetes	102584	3943
Hypertension	88726	21791
Handcap	108286	2241 (multi-level)

**Fig 6:**
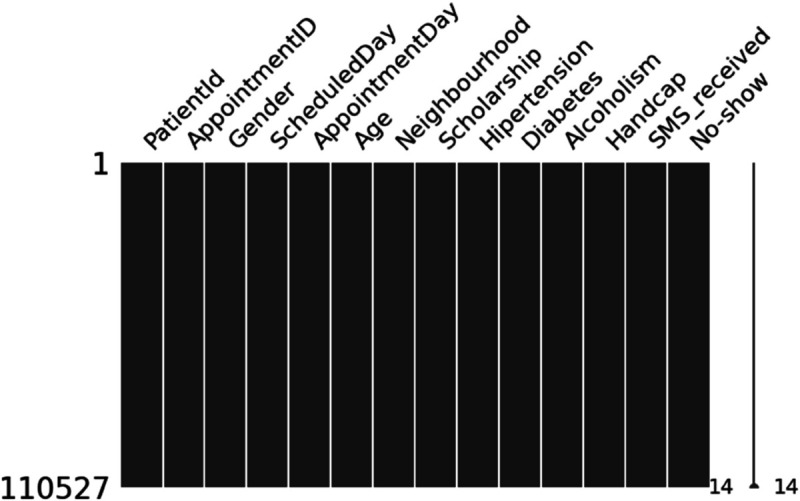
Missing value matrix confirming complete data coverage across all key features.

Fig 6 shows that there are no missing values in the dataset, which means the data is well-structured and consistent. Because of this, we do not need to use methods like filling in averages, interpolation, or dropping rows. This makes preprocessing easier and more reliable. It also helps ensure that the models are not affected by problems caused by missing data. Since both numerical and categorical features are complete, the later steps like encoding and scaling can be done smoothly.

**Fig 7:**
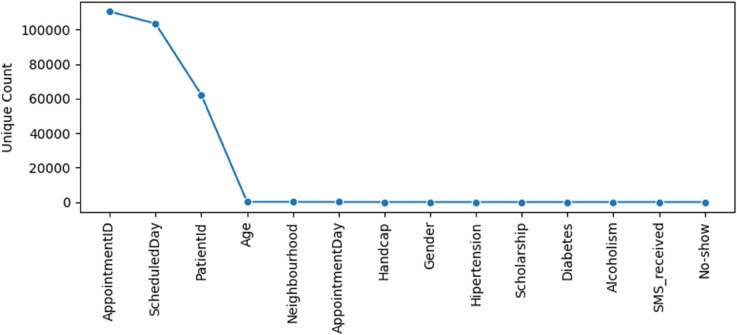
Unique values per feature.

Fig 7 shows that most columns in the dataset contain only a limited number of distinct values, making them well suited for conventional encodings such as binary or one-hot transformations. This contributes to a compact and stable feature space during preprocessing. By contrast, columns such as AppointmentID and PatientID exhibit extremely high cardinality and function primarily as identifiers rather than informative predictors; these fields are therefore excluded from modeling to avoid unnecessary sparsity and overfitting.

A similar consideration applies to the ScheduledDay field, which also contains a very large number of unique timestamp values. Instead of treating it as a categorical feature, the raw datetime information is decomposed into more interpretable and lower-cardinality temporal attributes, including waiting_days, scheduled_month, and scheduled_weekday. This transformation retains the meaningful temporal structure embedded in the original timestamp while ensuring the resulting variables are more compatible with downstream learning algorithms.

**Fig 8:**
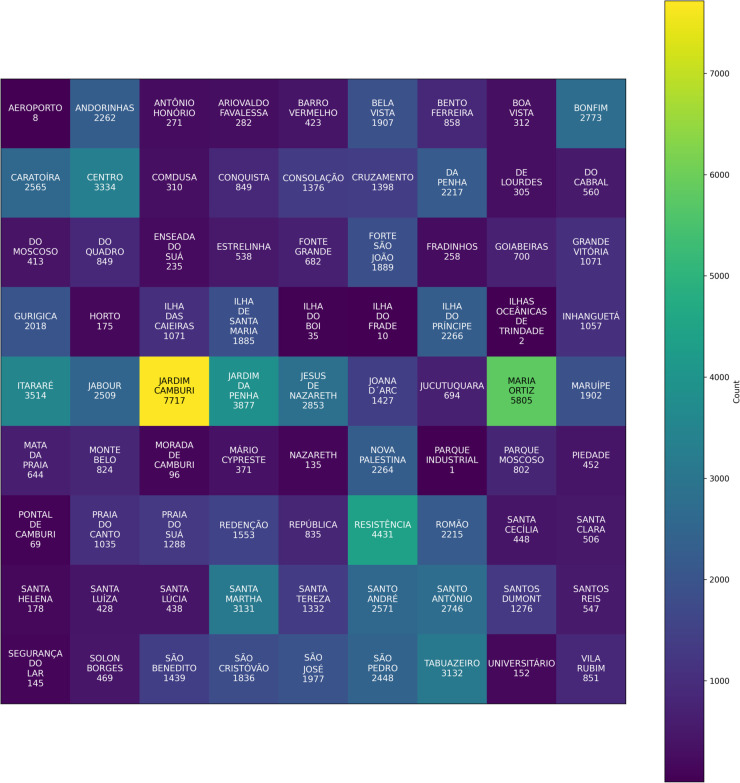
Heatmap of appointment counts across neighborhoods.

Fig 8 presents a heatmap summarizing appointment volumes across all neighborhoods. Unlike a traditional bar chart, this grid-based representation prevents x-axis overcrowding and offers a clearer comparison between locations with highly uneven population sizes or healthcare access patterns. Darker cells correspond to neighborhoods with higher appointment frequencies, highlighting substantial geographic variability in service demand.

Such spatial heterogeneity may reflect differences in population density, socioeconomic conditions, or the distribution of healthcare facilities. Neighborhoods with elevated appointment counts often correspond to areas with larger populations or greater reliance on public health services. Conversely, sparsely represented neighborhoods may indicate limited healthcare access or smaller local populations. Capturing these geographic disparities is essential for understanding the contextual factors that influence attendance patterns and for identifying potential structural biases within the dataset.

**Fig 9:**
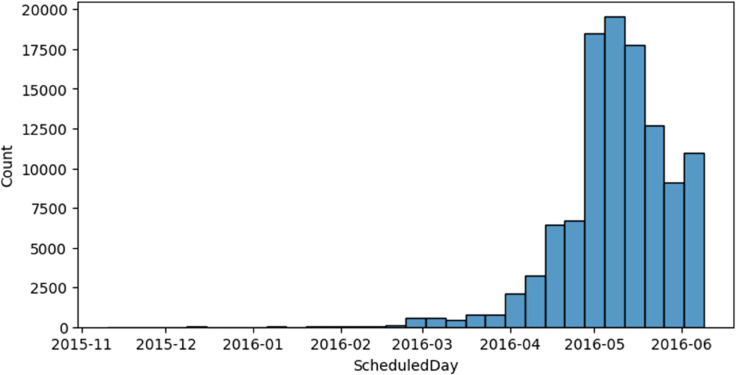
Scheduled appointments over time.

Fig 9 shows that the number of scheduled appointments increased sharply during April–May 2016. This rise could be due to seasonal reasons, like more people getting sick at that time of year, or it might be the result of changes in healthcare policies, such as a public health campaign or new scheduling rules. Identifying such temporal surges is important for understanding workload fluctuations and planning resource allocation in healthcare services.

**Fig 10:**
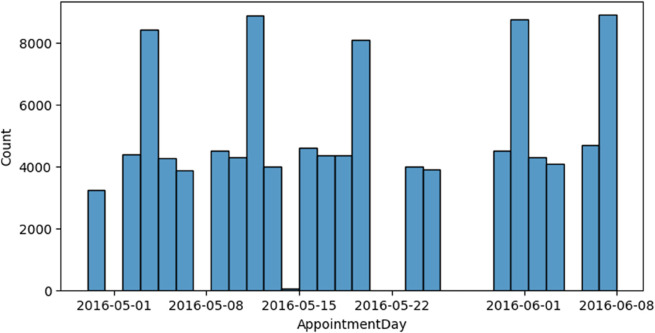
Appointment dates distribution.

Fig 10 shows a clear weekly pattern in when appointments actually took place. This regular cycle is likely due to how the clinics operate, for example, having fewer staff or being closed on weekends, as well as patients preferring certain days of the week for their visits. Understanding these cycles is essential for optimizing appointment availability, minimizing no-shows, and aligning service capacity with patient demand.

**Fig 11:**
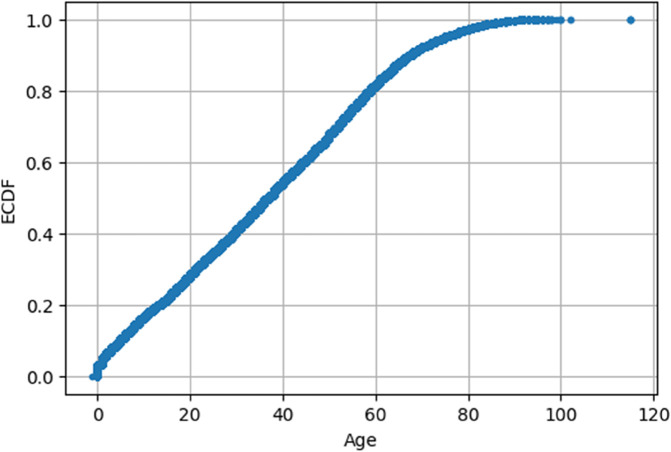
Age ECDF distribution.

The ECDF in Fig 11 confirms that a majority of patients are under the age of 60, with a long right tail extending to over 100. This right-skewed distribution implies that age is not uniformly distributed across the dataset. Such skewness, along with its potential correlation with chronic conditions and appointment behavior, suggests that age may serve as a valuable predictor in modeling patient attendance.

### 4.3 Feature engineering and selection

**Fig 12:**
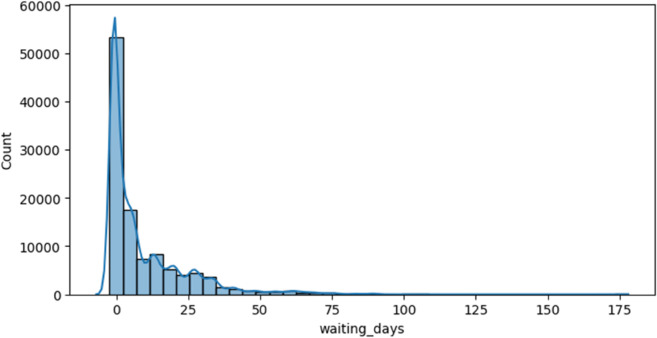
Waiting days distribution.

Fig 12 shows that most patients get appointments shortly after booking, while only a few have to wait a long time. Because of this uneven distribution, it may help to apply techniques like log scaling or grouping values into bins to make the data easier for models to learn from.

**Fig 13:**
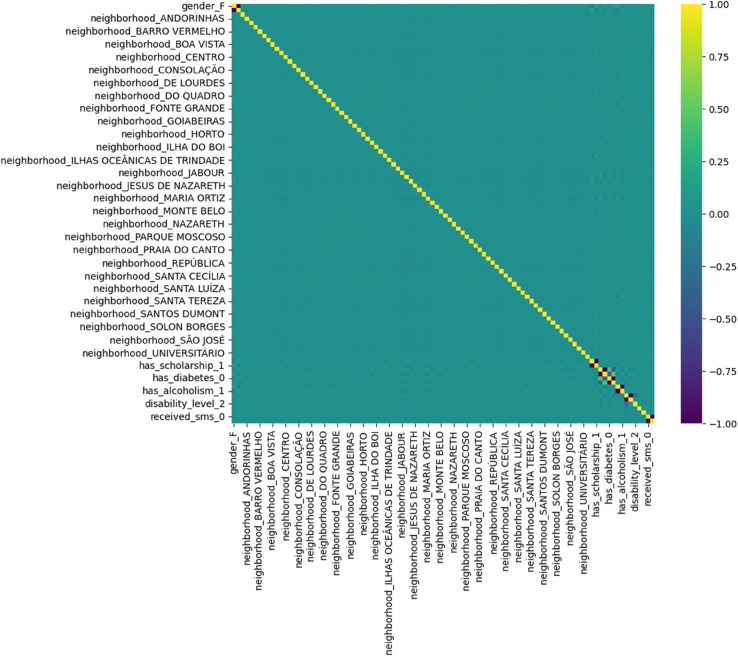
Correlation among encoded features. The feature encoding map used to generate the encoded matrix is provided in S2 Table.

Fig 13 reveals minimal correlation among the one-hot encoded categorical variables. This indicates that the encoded features are largely independent, thereby reducing the risk of multicollinearity and justifying their inclusion in the model without dimensionality reduction.

**Fig 14:**
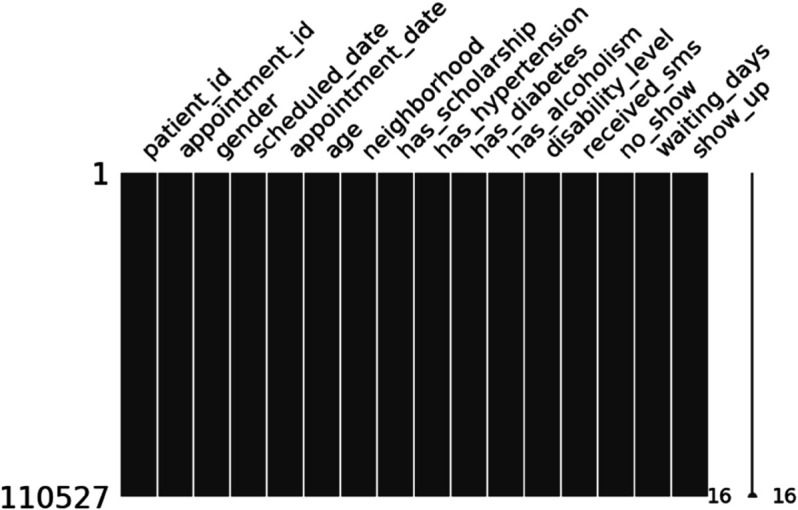
Missingness after engineering. The full missingness report is provided in S3 Table.

The matrix in Fig 14 shows that there are no missing values left after preprocessing. This means that the cleaning and transformation steps worked well, making the data complete and ready for modeling.

**Fig 15:**
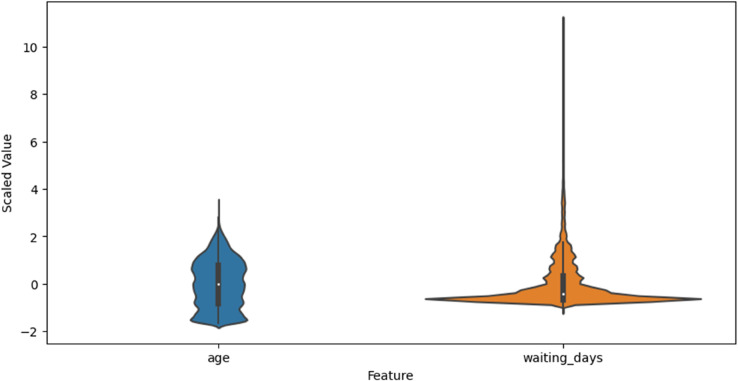
Scaled numeric features (violin plot).

Fig 15 shows the normalized distributions of key numerical features after scaling. Standardization centers each feature around zero with unit variance, which not only improves convergence speed for gradient-based algorithms but also ensures that features contribute proportionally to model decisions. The visualization also reveals differing spread and modality such as the long right tail in waiting_days which may influence how models weigh each feature. These transformations help make model training more stable and allow fair comparisons between features that originally had very different value ranges.

### 4.4 LLM fine-tuning evaluation

**Fig 16:**
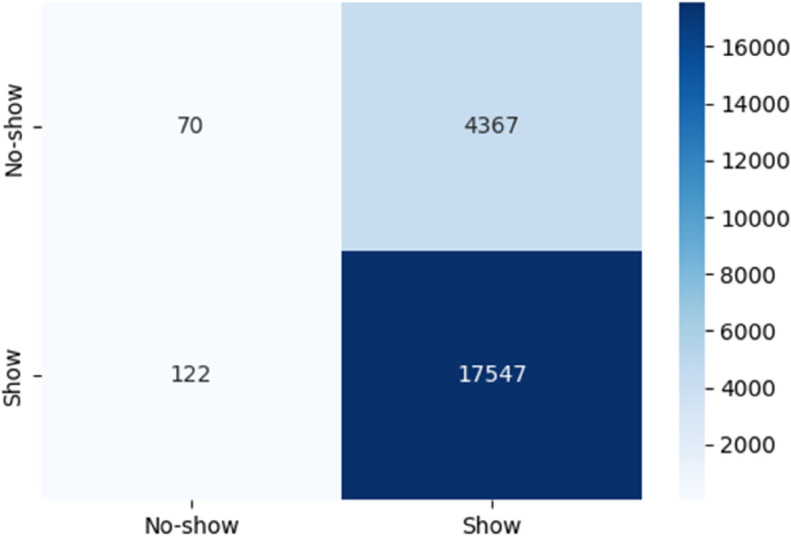
Confusion matrix of predictions.

Fig 16 illustrates the behavior of a transformer-based model when applied to the no-show dataset under an exploratory text-serialization setup. While the model captures the dominant Show class reasonably well, it performs poorly on the minority No-show class, as shown by the low true-negative count and large number of false negatives. This pattern reflects the well-known difficulty transformer models face when used directly for imbalanced tabular classification without specialized architectures or calibration. These exploratory findings further justify the decision to rely on structure-aware classical models in the main pipeline, as they achieve more stable and interpretable performance under the same class imbalance.

Table 12 reports the class support values used during evaluation. These counts provide context for the performance comparison in Table 9, illustrating the substantial class imbalance present in the dataset. The distribution highlights why minority-class performance remains challenging and why macro-averaged metrics must be interpreted alongside raw accuracy.

**Table 12:** Class distribution and support counts for the fine-tuned LLaMA 7B model.

Class	Support	Proportion (%)
No-show (0)	4437	20.1%
Show (1)	17669	79.9%
**Total**	22106	100%

**Fig 17:**
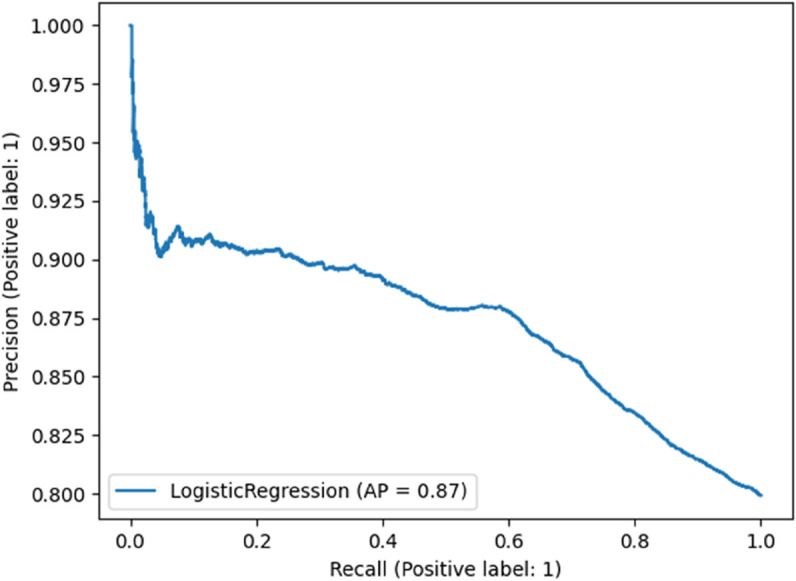
Precision–recall curve of the logistic regression classifier (AP = 0.87).

Fig 17 illustrates the precision–recall behavior obtained from an exploratory experiment using LLM-derived representations. The overall area under the curve (0.87) is largely driven by the model’s ability to recognize the majority Show class, which dominates the dataset. This high score therefore reflects class prevalence rather than meaningful improvements in minority detection. A closer inspection of the curve reveals that the approach still struggles to capture true No-show instances, a known limitation when generic language models are applied directly to imbalanced tabular data. These observations reinforce why the main pipeline relies on classical machine-learning models trained on the LLaMA-guided feature set for stable and interpretable predictive performance.

**Fig 18:**
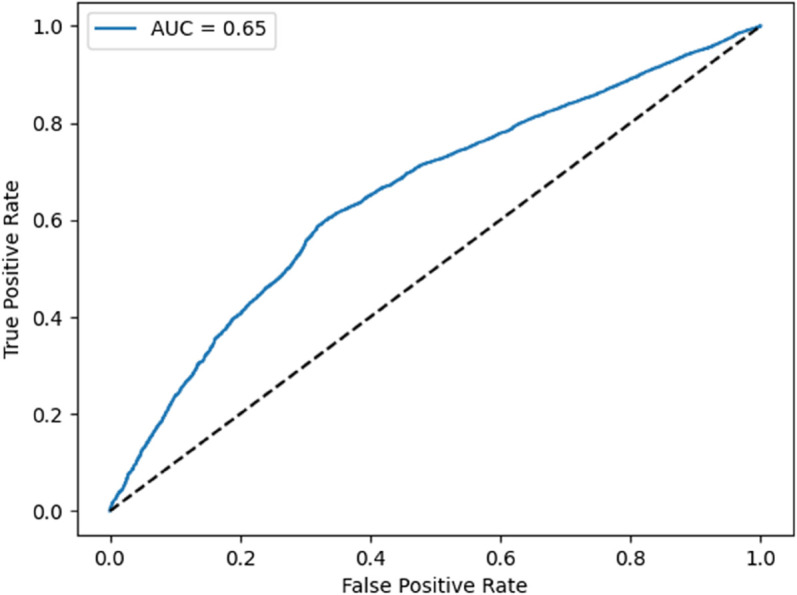
ROC curve with AUC = 0.65.

As shown in Fig 18, the ROC curve shows moderate discrimination ability with an AUC of 0.65. This is above random but far from ideal, underscoring the need for further tuning, especially for detecting minority-class no-shows.

**Fig 19:**
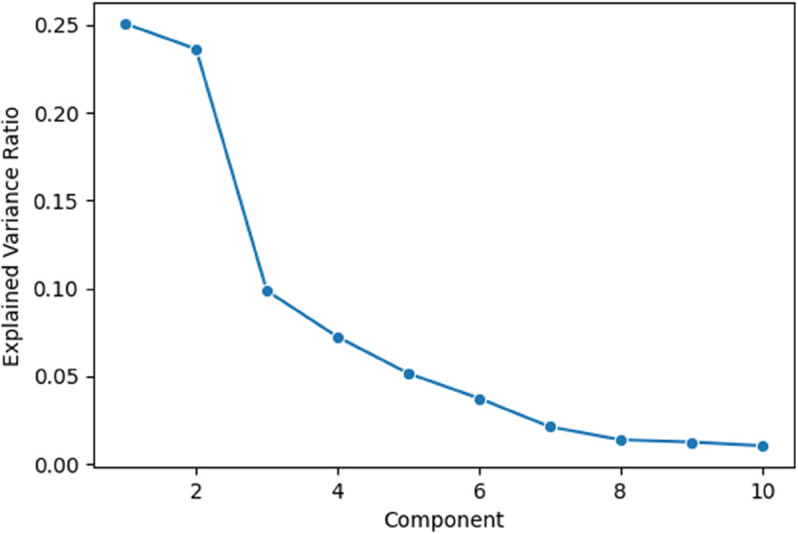
Explained variance per PCA component.

**Fig 20:**
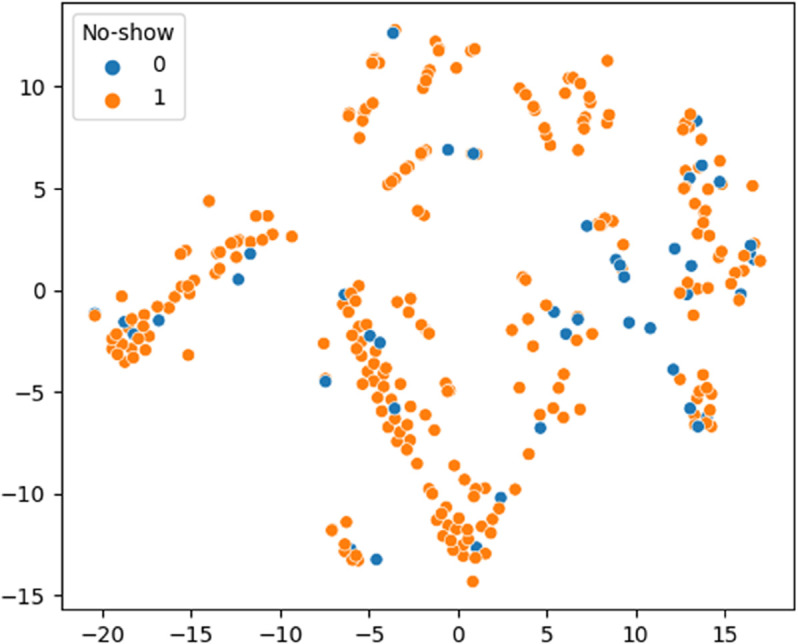
t-SNE visualization of LLaMA embeddings. Separation between ‘Show’ and ‘No-show’ is evident but not clean, indicating potential for further tuning.

**Latent embedding diagnostics.** Fig 19 shows how much variance each principal component captures from the LLM-generated embeddings. The first two components together explain nearly 50 percent of the total variation, meaning they preserve much of the original structure of the data. This makes them highly useful for visualizing patterns and relationships, while also reducing complexity for tasks like clustering and anomaly detection. As shown in Fig 20, the t-SNE projection of LLaMA embeddings reveals partially separated clusters for ‘Show’ and ‘No-show’ cases, indicating that the model captures label-relevant structure but still leaves room for further tuning.

Overall, the exploratory transformer-based model tends to favor the majority Show class, reflecting the underlying class imbalance rather than meaningful discriminative learning. Its difficulty in correctly identifying No-show cases highlights a limitation of applying generic language models directly to imbalanced tabular data. This observation reinforces the motivation for relying on classical structure-aware models in the main pipeline, which handle such distributions more robustly and offer interpretable decision structures. Techniques such as resampling, class weighting, or tabular-specific architectures would be necessary for LLM-based classifiers to perform competitively further underscoring why the final predictive results in this work are based on traditional machine learning models rather than LLMs.

### 4.5 Explainable AI via SHAP analysis

**Fig 21:**
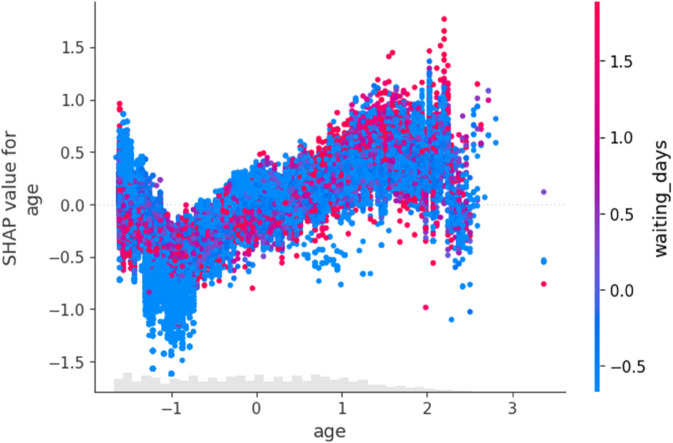
SHAP dependence plot for age.

Fig 21 illustrates how patient age affects model predictions with interaction from waiting time. The positive slope for mid-to-old age groups confirms the significance of age in no-show risk, especially when paired with longer delays, colored by waiting_days. This visualization shows a nonlinear relationship where higher age values contribute more positively to the model’s output, particularly when waiting_days are also high. Younger patients typically have lower SHAP values, indicating reduced model influence.

**Fig 22:**
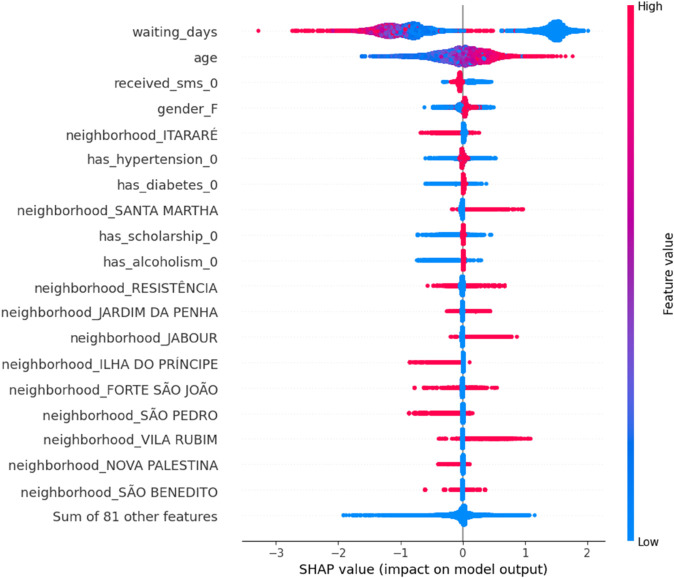
SHAP summary plot showing global feature importance and individual SHAP value distribution.

As shown in Fig 22, waiting_days and age emerge as top contributors to model output. High waiting times and advanced age generally increase the probability of a no-show, as evident from their rightward SHAP contributions. The spread also highlights the varying impact of neighborhoods and socio-demographic factors.waiting_days and age dominate model predictions. Red indicates high feature values, while blue indicates low.

**Fig 23:**
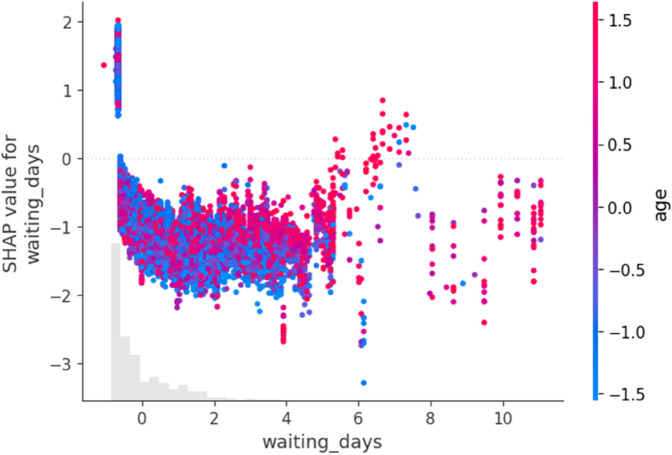
SHAP interaction plot between waiting_days and age.

Fig 23 shows that age and waiting time work together in affecting no-show risk. Older patients who are given appointments far in advance are more likely to miss them. This highlights how important it is to consider scheduling when caring for elderly patients. It shows that longer waiting periods combined with older age result in higher SHAP contributions and a higher predicted likelihood of no-show, while short waiting periods across ages exert a negative influence.

Table 13 quantitatively ranks features using mean absolute SHAP values, reaffirming the visual insights from Figs 22 and 21. Appointment delay and age are confirmed as the strongest drivers of model predictions. This table is derived from the SHAP feature ranking CSV and reflects the global influence of each predictor

**Table 13:** Top-ranked features by mean SHAP value (importance). The complete feature ranking is provided in S4 Table.

Feature	Mean SHAP Value
waiting_days	**0.241**
age	0.195
received_sms_0	0.082
gender_F	0.059
neighborhood_ITARARÉ	0.041
has_hypertension_0	0.038
has_diabetes_0	0.034
neighborhood_SANTA MARTHA	0.030

### 4.6 Profiling and resource usage

**Fig 24:**
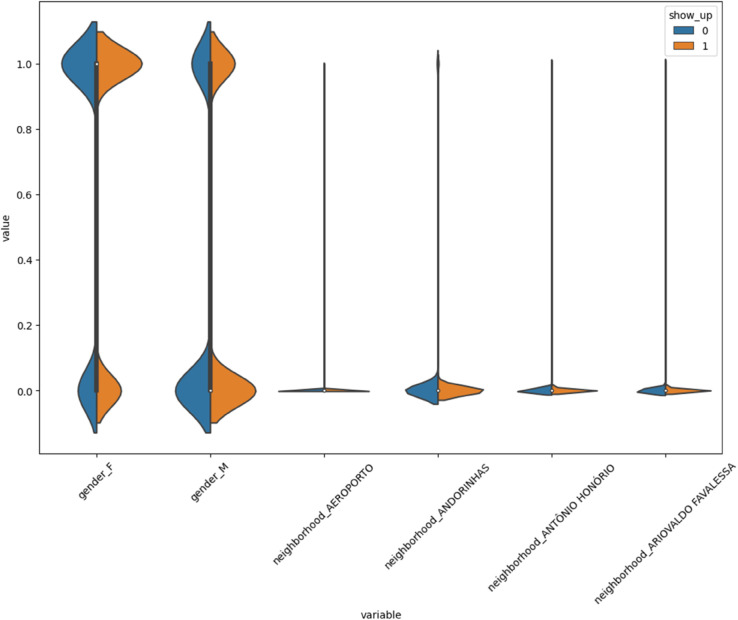
Violin plots by show-up status.

Fig 24 shows that some categorical features like gender and neighborhood have different patterns for patients who showed up versus those who didn’t. This suggests that where a patient lives and their demographic background may affect whether they attend their appointment.

**Fig 25:**
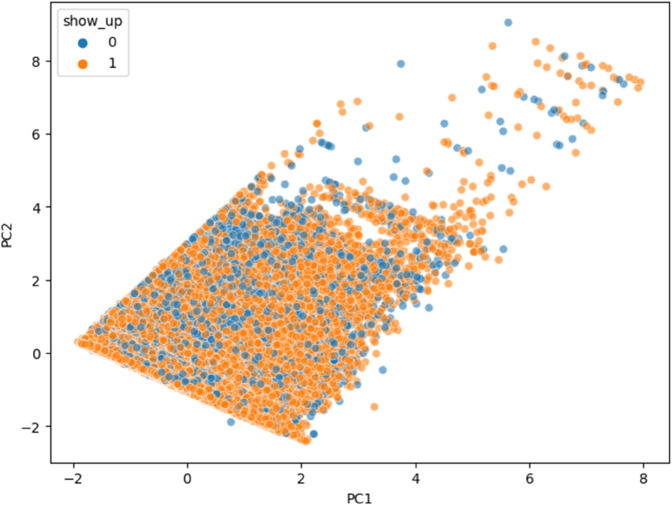
PCA scatterplot of features.

As shown in Fig 25, the PCA plot of the final features shows some separation between the no-show and show-up classes, especially along the first main direction (principal component). This suggests that the preprocessing and feature transformations helped bring out useful patterns in the data.

**Fig 26:**
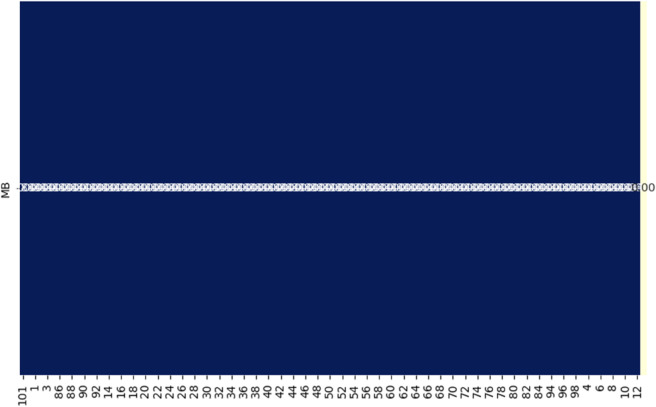
Feature-wise memory usage.

Fig 26 shows how much memory each feature uses. The one-hot encoded neighborhood features take up the most space, highlighting a common trade-off between detailed categorical granularity and efficient resource use. Compared to full AutoML frameworks such as Auto-Sklearn or H2O both of which maintain multiple candidate models, large search spaces, and repeated cross-validation artifacts in memory our pipeline remains considerably lighter. Because the proposed workflow does not perform exhaustive hyperparameter search or ensemble construction, the overall memory footprint stays bounded and predictable, with intermediate artifacts limited to preprocessing outputs and a single trained model. This makes the pipeline substantially more resource-efficient and suitable for local or constrained environments where AutoML systems may require significantly more RAM and compute to execute their search procedures.

## 5 Conclusion

This study has showcased a comprehensive and detailed summary of the pipeline that has analyzed and modeled the patient no-shows in medical appointments using the “Medical Appointment No Shows” dataset. Leveraging a local LLM for semantic feature enhancement, the pipeline systematically integrated domain-aligned metadata generation, automated type inference, and robust preprocessing. At the same time, the Detailed exploratory analysis has also helped us to reveal the key behavioral and demographic trends, notably the influence of waiting_days, age, and neighborhood-based socioeconomic factors.

This hybrid feature selection approach, by combining correlation analysis and SHAP-based model explainability, has been employed to reduce the dimensionality of the dataset and also preserve predictive power. Through detailed profiling and visualization, the refined dataset was validated for both memory efficiency and class separability using PCA.

The final framework ensures explainability, scalability, and resource efficiency, operating entirely within a local environment utilizing a Tesla P100 GPU. Visual summaries across each phase from ECDF plots to SHAP interaction diagrams affirm the interpretability of model behavior and feature impact.

Future work might incorporate the federated data from the multiple clinics, including the deeper socio-geographic context, and benchmarking of lightweight transformer-based predictors. This pipeline sets a strong foundation for trustworthy, interpretable, and locally deployable healthcare analytics.

### 5.1 Future work

Although the current study offers a robust and interpretable pipeline for predicting medical appointment no-shows using tabular data and a local LLM, several avenues remain for future enhancement:

- **Multi-Modal and Temporal Modeling:** By expanding this dataset to add more modalities like the clinical notes for each patient, lab results by the laboratory, or wearable sensor data, a comprehensive understanding of patient behavior could be achieved. Moreover, incorporating temporal features such as appointment history and chronic condition trajectories can capture longitudinal dynamics that static models may miss.
- **Federated and Edge-Based Deployment:** To ensure privacy-preserving and scalable deployment in healthcare settings, future work can also leverage the state-of-the-art federated learning to train across multiple institutions without sharing raw data for privacy concerns. Additionally, optimizing the pipeline for edge deployment on low-power hardware like the Jetson Nano or Coral TPU could enable real-time inference in remote or under-resourced clinics.
- **Domain-Adaptive LLM Enhancements:** While the current setup simulates a general-purpose local LLaMA-7B model, future research could explore domain-specific LLM fine-tuning, prompt engineering, or retrieval-augmented generation (RAG) to further enhance feature engineering, metadata synthesis, and interpretability.

These directions would strengthen both the operational scalability and real-world clinical applicability of the proposed system.

## Supporting information

S1 Table Categorical variable summary. Counts for all categorical variables after preprocessing, supporting Table 11.
(CSV)

S2 Table Feature encoding dictionary. One-hot and label-encoding mapping from raw variables to encoded feature columns used for Fig 13.
(CSV)

S3 Table Post-engineering missing-data report. Variable-level missingness statistics after feature engineering, supporting Fig 14.
(CSV)

S4 Table SHAP feature importance ranking. Full ranked list of mean SHAP values for all features, supporting Table 13.
(CSV)
